# In Vitro *N*-Glycan Mannosyl-Phosphorylation of a Therapeutic Enzyme by Using Recombinant Mnn14 Produced from *Pichia pastoris*

**DOI:** 10.4014/jmb.2010.10033

**Published:** 2020-10-28

**Authors:** Ji-Yeon Kang, Hong-Yeol Choi, Dong-Il Kim, Ohsuk Kwon, Doo-Byoung Oh

**Affiliations:** 1 Korea Research Institute of Bioscience and Biotechnology (KRIBB), Daejeon 344, Republic of Korea; 2 Department of Biological Engineering, Inha University, Incheon 1, Republic of Korea; 3 Biosystems and Bioengineering Program, University of Science and Technology (UST), Daejeon 411, Republic of Korea

**Keywords:** Mannosyl-phosphorylation, Mannose-6-phosphate, Mnn14, enzyme replacement therapy, lysosomal storage disease

## Abstract

Enzyme replacement therapy for lysosomal storage diseases usually requires recombinant enzymes containing mannose-6-phosphate (M6P) glycans for cellular uptake and lysosomal targeting. For the first time, a strategy is established here for the in vitro mannosyl-phosphorylation of high-mannose type *N*-glycans that utilizes a recombinant Mnn14 protein derived from *Saccharomyces cerevisiae*. Among a series of N-terminal- or C-terminal-deleted recombinant Mnn14 proteins expressed in *Pichia pastoris*, rMnn14_77-935_ with deletion of N-terminal 76 amino acids spanning the transmembrane domain (46 amino acids) and part of the stem region (30 amino acids), showed the highest level of mannosyl-phosphorylation activity. The optimum reaction conditions for rMnn14_77-935_ were determined through enzyme assays with a high-mannose type *N*-glycan (Man_8_GlcNAc_2_) as a substrate. In addition, rMnn14_77-935_ was shown to mannosyl-phosphorylate high-mannose type Nglycans (Man_7-9_GlcNAc_2_) on recombinant human lysosomal alpha-glucosidase (rhGAA) with remarkably high efficiency. Moreover, the majority of the resulting mannosyl-phosphorylated glycans were bis-form which can be converted to bis-phosphorylated M6P glycans having a superior lysosomal targeting capability. An in vitro *N*-glycan mannosyl-phosphorylation reaction using rMnn14_77-935_ will provide a flexible and straightforward method to increase the M6P glycan content for the generation of “Biobetter” therapeutic enzymes.

## Introduction

**Fig. 1:**
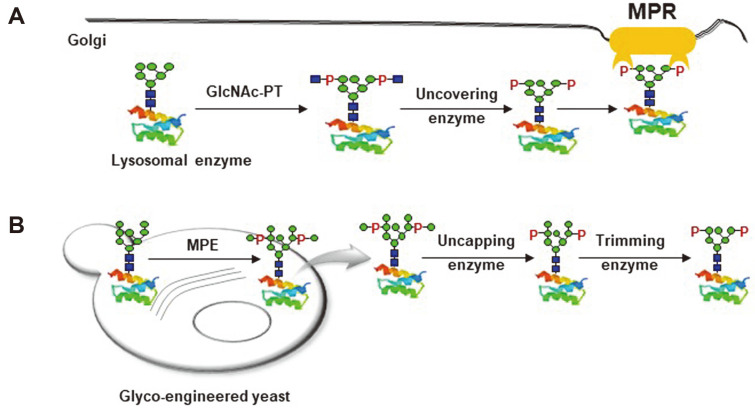
Schematic representations of M6P glycan generations. (**A**) In mammalian Golgi, GlcNAc-1-phosphotransferase recognizes lysosomal enzymes and transfers the GlcNAc-1-phosphate moiety of UDP-GlcNAc to the mannose residues of the high-mannose type *N*-glycans on enzymes. Then, an uncovering enzyme removes the outer GlcNAc to leave a phosphate group linked to a mannose residue. The resulting M6P glycans are recognized by MPRs on the Golgi membrane for traffic to the lysosome. (**B**) Recombinant proteins containing a large amount of mannosyl-phosphorylated glycans can be produced in glyco-engineered yeast overexpressing MPEs. The mannosyl-phosphorylated glycans on the secreted proteins can be uncapped and trimmed in vitro by using an uncapping enzyme and a trimming mannosidase, which generates M6P glycans for MPR targeting. Monosaccharide symbols follow the Symbol Nomenclature for Glycans(SNFG) system, details of which are found at NCBI: green circle, mannose; blue square, GlcNAc; P, phosphate [7].

Lysosomal storage diseases (LSDs), a group of inherited diseases, are caused by hydrolase deficiency, which results in the accumulation of undigested metabolites in the lysosome. Enzyme replacement therapy (ERT) with an intravenous injection of a therapeutic recombinant enzyme is the prevalent option for treating LSDs [1]. Most therapeutic enzymes, except for those associated with Gaucher disease, require mannose-6-phosphate (M6P) glycans for efficient lysosomal targeting [1-3]. In the mammalian Golgi apparatus, M6P glycans are generated by a two-step process (Fig. 1A) [1]. First, GlcNAc-1-phosphotransferase adds GlcNAc-1-phosphate to the mannose residue of high-mannose type glycans. Then, the uncovering enzyme removes the outer GlcNAc to expose a phosphate group linked to the mannose residue, resulting in phosphate-6-*O*-mannose structure called M6P. Lysosomal enzymes containing M6P glycans are recognized by M6P receptors (MPRs) on the Golgi membrane. The resulting MPR-enzyme complexes are delivered to endosomes. As endosome maturation accompanies the decrease in the pH level, lysosomal enzymes are released from MPRs and go alone to the lysosome. Some enzymes escaping from this MPR pathway are secreted into the extracellular space. These secreted enzymes can be recaptured by the MPRs on the plasma membrane and delivered to the lysosome through MPR-mediated endocytosis. ERTs exploit this “MPR-based recapture pathway” for the cellular uptake and lysosomal targeting of infused therapeutic enzymes.

Because the M6P glycan content is a key factor determining the therapeutic efficacy of ERTs, glyco-engineering strategies for increasing the M6P glycan content have been designed to produce “Biobetter” enzymes for LSDs [1]. First, a strategy to conjugate synthetic M6P glycans to recombinant therapeutic enzymes was developed and successfully employed for recombinant human lysosomal alpha-glucosidase (rhGAA, brand name “Myozyme”) for the treatment of Pompe disease. Conjugation of synthetic M6P glycans to rhGAA increased its binding affinity to MPRs and therefore improved the therapeutic efficacy significantly in a Pompe disease mouse model [4-6]. Second, a strategy involving the heterologous expression of target therapeutic enzymes having mannosyl-phosphorylated glycans and subsequent in vitro enzymatic uncapping and trimming reactions has been established [3]. rhGAA containing mannosyl-phosphorylated glycans was expressed in a glyco-engineered yeast strain by deletion of its yeast-specific hyper-mannosylation pathway gene and introduction of a mannosyl-phosphorylation enzyme (MPE) gene (Fig. 1B). After secretory expression in this yeast, the mannosyl-phosphorylated glycans of rhGAA were converted to M6P glycans through in vitro uncapping and trimming reactions [3].

Mnn14 and YlMpo1 have been found to be the main MPEs in *Saccharomyces cerevisiae* and *Yarrowia lipolytica*, respectively, in our recent study involving gene disruptions and complementation experiments [7, 8]. If a recombinant MPE is available for an in vitro reaction, it will allow the development of another strategy to produce a “Biobetter” enzyme with a high content of M6P glycans through a combination of mannosyl-phosphorylation, uncapping, and trimming reactions. Here, for the first time, we report the production of a soluble recombinant MPE capable of adding mannosyl-phosphate efficiently to the high-mannose type *N*-glycans of a therapeutic enzyme.

## Materials and Methods

### Construction of Expression Vectors

The microbial strains and plasmids used in this study are listed in Table S1. To construct vectors expressing recombinant MPEs, the corresponding DNA fragments of Mnn14 and YlMpo1 genes were amplified by polymerase chain reaction (PCR) using pYEp352-Mnn14 [8] and pYEp352-YlMpo1 [7] as templates, respectively, with primers (Table S2). The PCR products were cloned into the pPIZαA vector using EZ-Fusion cloning kits (Enzynomics, Korea) and were propagated in *Escherichia coli* DH5α.

### Secretory Expressions in *P. pastoris*

The recombinant expression vectors were transformed into the *P. pastoris* PPS9016 strain by electroporation, and the transformants were selected on YPD (1% yeast extract, 2% Bacto peptone, 2% glucose) agar plates containing 0.1 mg/ml Zeocin. A single colony was initially cultured in 1 ml YPD medium containing 0.1 mg/ml Zeocin for 16 h, which was then transferred to flasks containing 10 ml BMMY (1% yeast extract, 2% peptone, 100 mM potassium phosphate, pH 6.0, 1.34% yeast nitrogen base, 0.00004% biotin, 0.5% MeOH) medium and cultured for two days at 30°C with shaking at 200 rpm with the addition of 0.05 ml of 0.5% methanol every 24 h for the induction of recombinant MPE. To choose the best clones secreting each recombinant MPE, 10 μg of total protein in the culture supernatants was separated by 10% sodium dodecyl sulphate-polyacrylamide gel electrophoresis (SDS-PAGE) and then transferred to a PVDF membrane for a Western blot analysis using an anti-Mnn14, anti-YlMpo1, or anti-His-tag antibody. Here, anti-Mnn14 and anti-YlMpo1 antibodies were generated using the synthesized peptides of Mnn14 and the denatured YlMpo1 protein expressed in *E. coli*, respectively (Koma Biotech, Korea).

### Mannosyl-Phosphorylation Assay Using a DNA Sequencer

In order to compare the enzyme activity of the secreted recombinant MPEs, mannosyl-phosphorylation assays were conducted using 5 μg of total protein obtained from the culture supernatants. Unless specifically described in the text, the assay buffer contained 50 μl of 50 mM Tris-HCl (pH 7.5), 10 mM MnCl_2_, 2 mM GDP-mannose (donor substrate), 5 μM M8-APTS (acceptor substrate), and 0.5 mM deoxymannojirimycin. This reaction was typically performed at 30°C for 10 min and was terminated by boiling. The reaction products were analyzed using a DNA sequencer based on a published protocol [9]. APTS-labeled glycans were loaded onto an ABI 3130 sequencer (Applied Biosystems, USA) equipped with a standard 36 cm capillary array filled with the POP-7 polyacrylamide linear polymer. The resulting electropherogram was analyzed with the GeneMapper software package (Applied Biosystems). To determine the specific enzyme activity (pmol/min/mg), the contents of mannosyl-phosphorylated glycans were calculated based on the normalized ratio of the corresponding peak area [100 x (the areas of mannosyl-phosphorylated glycan peaks)/(total areas of all identified peaks)], as described previously [8].

### Purification of rMnn14_77-935_

The *P. pastoris* harboring pPrMnn14_77-935_ was cultured in the BMMY medium for 48 h at 30°C with shaking at 200 rpm, as described in the subsection entitled ‘Secretory Expressions in *P. pastoris*’. The culture supernatant was obtained by centrifugation at 8,000 g for 20 min and concentrated by the ultracentrifugal ﬁltration using a YM50 membrane (Millipore, USA). The concentrate was then dialyzed against a binding buffer (20 mM Tris-HCl, pH 7.5). The dialyzed sample was loaded onto a Q-HP column (GE Healthcare) pre-equilibrated with the binding buffer. After a harsh washing step using more than 10 column volumes of the binding buffer, elution was conducted by a linear gradient of 0 – 1 M NaCl in the binding buffer for 50 min. All fractions were collected and analyzed by SDS-PAGE and MPE assays.

### Effects of the pH, Temperature, and Metal Ions on the Mannosyl-Phosphorylation Activity of rMnn14_77-935_

To examine the effects of the pH on the mannosyl-phosphorylation activity of rMnn14_77-935_, the enzyme activity was measured between pH 4.0 and 8.0 using 50 mM sodium acetate buffer (pH 4.0-5.5), 50 mM sodium phosphate buffer (pH 6.0-7.5), and 50 mM Tris buffer (pH 6.0-8.0). To determine the optimal temperature, the reaction temperature was varied from 25°C to 50°C. To evaluate the effects of metal ions, MPE assays were carried out in the presence of a 10 mM final concentration of various metal ions (MnCl_2_, MgCl_2_, CaCl_2_, FeCl_3_, CuSO_4_, or ZnSO_4_) or ethylenediaminetetraacetic acid (EDTA). To find the optimum concentration, the MPE assays were performed with 1, 2, 5, 10, or 20 mM of MnCl_2_.

### Preparation of rhGAA Containing High-Mannose Type *N*-Glycans

The rhGAA-containing high-mannose type *N*-glycans were prepared as described previously [10]. Briefly, they were produced in transgenic rice cell suspension cultures via a treatment with two mannosidase inhibitors (kifunensine and swainsonine) to generate high-mannose type *N*-glycans (Man_7-9_GlcNAc_2_) instead of plant-specific complex type glycans. His-tagged rhGAA was purified from the suspension cultured media using Ni-NTA Superflow cartridges (Qiagen, USA).

### In Vitro Mannosyl-Phosphorylation of rhGAA

For in vitro mannosyl-phosphorylation, 5 μg of rhGAA was incubated in 50 μl of a reaction buffer containing 50 mM Tris-HCl buffer (pH 7.5), 10 mM MnCl_2_, 2 mM GDP-mannose, 0.5 mM deoxymannojirimycin, and rMnn14_77-935_ (0.5 mg/ml). After incubation at 30°C for 24 h, the reaction was terminated by boiling for 5 min.

### *N*-Glycan Analysis of rhGAA

*N*-glycans obtained from rhGAA were analyzed using high-performance liquid chromatography (HPLC), as described previously [11]. Briefly, *N*-glycans were released by a PNGase A treatment and purified by solid-phase extraction using graphitized carbon. The purified glycans were labeled with 2-aminobenzamide (2-AA) for fluorescent detection during the HPLC analysis. The 2-AA-labeled glycans were separated on a Shodex Asahipak NH2P-50 4E (4.6 mm × 250 mm) column (Showa Denko, Japan) using a Waters Alliance system equipped with a Waters 2475 fluorescence detector (Waters, USA). Solvent A consisted of acetonitrile containing 2% acetic acid and 1% tetrahydrofuran. Solvent B consisted of 10% acetic acid, 6% triethylamine, and 1% tetrahydrofuran in water. Elution was carried out with a linear gradient from 90% A and 10% B to 10% A and 90% B at a flow rate of 1 ml/min for 70 min at 50°C. The fluorescence levels of the AA-labeled glycans were monitored with excitation at 360 nm and emission at 425 nm. After the HPLC glycan profile analysis, each glycan peak was collected and analyzed using a Microflex MALDI-TOF mass spectrometer (Bruker Daltonik, GmbH, Germany), as described previously [11]. Briefly, 2-AA-labeled glycans were spotted on a MALDI MSP96 polished steel chip (Bruker Daltonik). A matrix solution of 6-Aza-2-thiothymin/2,5-dihydroxybenzoic acid was then added and dried in air. Because mannosyl-phosphorylated glycans have negative charges in their phosphate group, all mass spectra were acquired in a linear negative ion mode.

## Results

### Secretory Expressions of Recombinant Mnn14 and YlMpo1 Proteins

Our previous results showed that YlMpo1 and Mnn14 have strong mannosyl-phosphorylation activity through complementation experiments in glyco-engineered *S. cerevisiae* [7, 8]. However, the corresponding in vitro mannosyl-phosphorylation reactions have yet to be shown because they are type II membrane proteins residing in the Golgi apparatus, meaning that soluble forms cannot be obtained. The N-terminal transmembrane domains of YlMpo1 and Mnn14 were replaced with the α-mating factor (α-MF) signal sequence in the pPIZαA vector for secretory expression in *P. pastoris*. When the constructed pPrMnn14_47-935_ and pPrYlMpo1_36-644_ vectors were transformed to *P. pastoris* and subjected to Western blot analyses using an anti-Mnn14 antibody and an anti-YlMpo1 antibody, respectively, many clones secreting rMnn14_47-935_ were found (Fig. S1A), whereas there were no clones secreting rYlMpo1_36-644_ (data not shown). Further, we constructed the vectors for the secretory expression of C-terminal His-tagged rMnn14_47-935_-H_6_ (pPrMnn14_47-935_-H_6_) and rYlMpo1_36-644_-H_6_ (pPrYlMpo1_36-644_-H_6_). Likewise, clones secreting rMnn14_47-935_-H_6_ were found (Fig. S1B), whereas there were no clones secreting rYlMpo1_36-644_-H_6_ (data not shown). Therefore, we focused on the secretory expression of recombinant Mnn14 proteins.

**Fig. 2:**
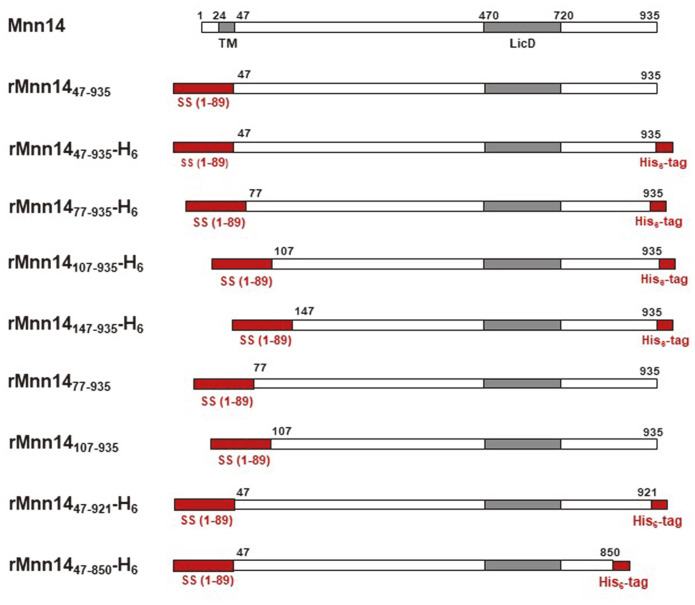
Schematic representations of various recombinant Mnn14 proteins. Mnn14 is a type II membrane protein that contains a transmembrane (TM) domain (24-46 residue) and LicD domain (470-720 residues) found in the LICD protein family (PF04991), a member of the nucleotidyltransferase fold proteins [8]. Recombinant Mnn14 (rMnn14) proteins were basically designed to be expressed with the mating factor α signal sequence (1-89) instead of the TM domain for secretion, in some cases with additional N-terminal or C-terminal deletions with or without C-terminal His-tag.

Because removal of the N-terminal or C-terminal part was reported to improve the enzyme activity in several glycosyltransferases [12], we constructed a series of vectors expressing recombinant Mnn14 proteins with N-terminal and C-terminal deletions (Fig. 2 and Table S1). Three vectors with N-terminal deletions of 76, 106, or 146 amino acids and the C-terminal addition of His-tag were constructed; pPrMnn14_77-935_-H_6_, pPrMnn14_107-935_-H_6_, and pPrMnn14_147-935_-H_6_ were designed to express rMnn14_77-935_-H_6_, rMnn14_107-935_-H_6_, and rMnn14_147-935_-H_6_, respectively. Two vectors with C-terminal deletions of 14 or 85 amino acids and the addition of His-tag were also constructed: pPrMnn14_47-921_-H_6_ and pPrMnn14_47-850_-H_6_ to express rMnn14_47-921_-H_6_ and rMnn14_47-850_-H_6_, respectively. *P. pastoris* clones secreting recombinant Mnn14 with N-terminal deletions (rMnn14_77-935_-H_6_, rMnn14_107-935_-H_6_, and rMnn14_147-935_-H_6_) were found (Figs. S1C-S1E), whereas clones secreting recombinant Mnn14 with C-terminal deletions (rMnn14_47-921_-H_6_ and rMnn14_47-850_-H_6_) were not detected (data not shown). These results indicate that the removal of the C-terminal part has a detrimental effect on the secretory expression of Mnn14.

Because the C-terminal part was suggested to be critical for maintaining the protein structure of some glycosyltrnsferases [12], we wondered whether C-terminal His-tag interferes with the enzyme activity of rMnn14. In order to test this possibility, two more vectors expressing recombinant Mnn14 proteins without C-terminal His-tag were constructed: pPrMnn14_77-935_ and pPrMnn14_107-935_ to express rMnn14_77-935_ and rMnn14_107-935_, respectively (Figs. 2G and 2H).

### Comparison of Mannosyl-Phosphorylation Activities

**Fig. 3:**
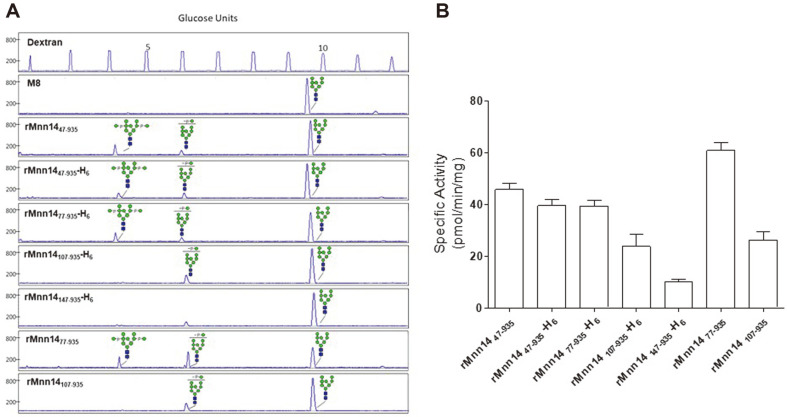
Mannosyl-phosphorylation activity analysis. (**A**) An APTS-labeled Man_8_GlcNAc_2_ glycan (M8) was used as a substrate for a mannosyl-phosphorylation analysis using a DNA sequencer. Mono-mannosyl-phosphorylated (ManP-Man_8_GlcNAc_2_) and/or bis-mannosyl-phosphorylated glycans (Man_2_P_2_-Man_8_GlcNAc_2_) are generated by the reaction using rMnn14_47-935_, rMnn14_47-935_-H_6_, rMnn14_77-935_-H_6_, rMnn14_107-935_-H_6_, rMnn14_77-935_, and rMnn14_107-935_ but not rMnn14_147-935_-H_6_. A dextran reference chromatogram is presented in the first panel to display the glucose units. Symbols are identical to those used in Fig. 1. (**B**) The specific enzyme activity (pmol/min/mg) was determined based on the contents of mannosyl-phosphorylated glycans calculated from the normalized ratio of the corresponding peak area [100 × (the areas of mannosyl-phosphorylated glycan peaks)/(total areas of all identified peaks)]. The quantified data represent the averages of three replicated experiments with standard deviations.

Clones secreting the largest amounts of rMnn14 variants were selected based on the Western blot analysis (Figs. S1A-S1G). The specific enzyme activities (pmol/min/mg) for mannosyl-phosphorylation in the culture supernatants of selected clones were then analyzed using a DNA sequencer method. Four recombinant enzymes (rMnn14_47-935_, rMnn14_47-935_-H_6_, rMnn14_77-935_-H_6_, and rMnn14_77-935_) converted the Man_8_GlcNAc_2_ glycan to the mono-mannosyl-phosphorylated (ManP-Man_8_GlcNAc_2_) and bis-mannosyl-phosphorylated forms (Man_2_P_2_-Man_8_GlcNAc_2_) (Fig. 3A). N-terminal deletion of 106 amino acids (rMnn14_107-935_-H_6_ and rMnn14_107-935_) led to the loss of the bis-mannosyl-phosphorylated form. The mannosyl-phosphorylation activity nearly disappeared upon N-terminal deletion of 146 amino acids (rMnn14_147-935_-H_6_). Fig. 3B clearly shows that rMnn14_77-935_ has the highest level of mannosyl-phosphorylation activity, suggesting that the deletion of the N-terminal 76 amino acids improves the activity of recombinant Mnn14. Notably, each of the rMnn14_47-935_, rMnn14_77-935_, and rMnn14_107-935_ samples showed higher activity than the corresponding variant with C-terminal His-tag, suggesting that the C-terminal His-tag interferes with the mannosyl-phosphorylation activity.

### Optimal Reaction Conditions of Purified rMnn14_77-935_ for Mannosyl-Phosphorylation

Because rMnn14_77-935_ has the best specific activity, it was purified from the culture supernatant. Briefly, after the concentration and dialysis of the culture supernatant against a binding buffer, rMnn14_77-935_ was purified by means of anion-exchange chromatography. The purified rMnn14_77-935_ was detected at approximately 100 kDa via the SDS-PAGE (Fig. S1H).

**Fig. 4:**
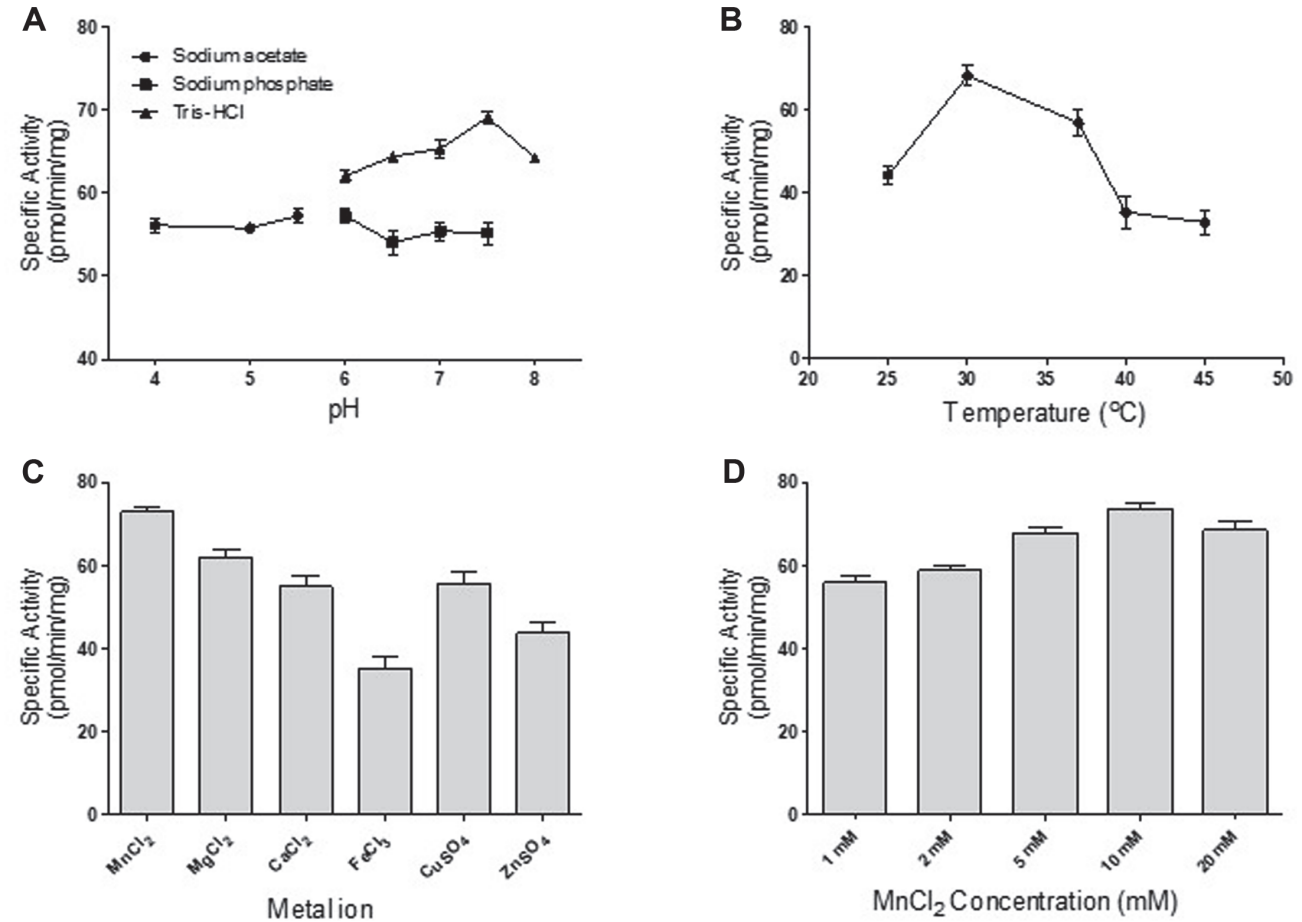
Optimal reaction conditions of rMnn14_77-935_ for in vitro mannosyl-phosphorylation. (**A**) The optimum pH for the rMnn14_77-935_ reaction was determined using three kinds of buffers; 50 mM of sodium acetate (pH 4.0-5.5), 50 mM of sodium phosphate (pH 6.0-7.5), and 50 mM of Tris-HCl (pH 6.0-8.0). (**B**) The optimum temperature was selected from 25°C to 50°C. (**C**) The metal ion effect was investigated to find the best metal ion. (**D**) The optimum concentration of MnCl_2_ was examined. The quantified data represent the averages of three replicated experiments with standard deviations.

We performed a series of experiments to find the optimal reaction conditions for the mannosyl-phosphorylation activity of purified rMnn14_77-935_. To find the best pH, an enzyme assay was performed under different pH levels ranging from pH 4.0 to pH 8.0. With Tris-HCl buffer ranging from pH 6.0 to pH 8.0, the highest activity was observed at pH 7.5. However, the activity level at the same pH decreased sharply when using sodium phosphate as a buffer. In addition, the activity levels at different pH values in Tris-HCl buffer were found to be higher than those when sodium acetate or sodium phosphate was used as a buffer (Fig. 4A). It was speculated that the composition of the buffer is also importantly related to the activity of the enzyme. The maximum level of enzyme activity was found at 30°C, and the activity rapidly decreased after 40°C (Fig. 4B). Among various metal ions (MnCl_2_, MgCl_2_, CaCl_2_, FeCl_3_, CuSO_4_, and ZnSO_4_) examined at 10 mM with regard to their effect on the enzyme activity of rMnn14_77-935_, MnCl_2_ was found to be best, and its optimal concentration was determined to be 10 mM (Figs. 4C and 4D). Because the metal chelator EDTA completely abolished enzyme activity (data not shown), the existence of metal ions appeared to be essential for mannosyl-phosphorylation activity. Based on these experiments, the optimum reaction condition was set to 50 mM Tris-HCl buffer (pH 7.5), 30°C, and 10 mM MnCl_2_.

### In Vitro Mannosyl-Phosphorylation of *N*-Glycans on rhGAA

Here, rhGAA was selected as a substrate for mannosyl-phosphorylation because it is associated with low therapeutic efficacy stemming from its low M6P content. The M6P content values of rhGAA were reported to be 0.7 to 1.3 mol/mol enzyme [13-14]. Myozyme, the rhGAA used for the treatment of Pompe disease, is produced from Chinese hamster ovary (CHO) cells. Out of seven N-glycosylation sites of Myozyme, five sites were occupied with complex type *N*-glycans, and only two sites (Asn140 and Asn470) were occupied with M6P glycans [13]. Because MPEs can mannosyl-phosphorylate high-mannose type *N*-glycans, rhGAA containing high-mannose type *N*-glycans was required for an in vitro mannosyl-phosphorylation experiment. Therefore, we prepared rhGAA containing high-mannose type *N*-glycans using a previously described method [10].

**Fig. 5:**
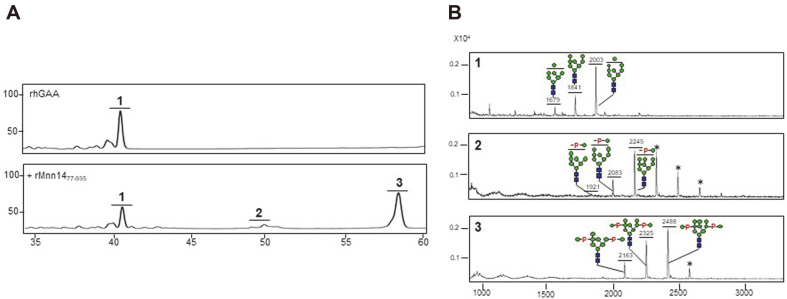
Generation of mannosyl-phosphorylated glycans on rhGAA by an in vitro reaction using rMnn14_77-935_. (**A**) The *N*-glycans released from rhGAA were analyzed in terms of their HPLC glycan profile (1). After the rMnn14_77-935_ reaction, the HPLC glycan profile analysis demonstrated the generation of mono-mannosyl-phosphorylated (2) and bismannosyl- phosphorylated glycan peaks (**3**). (B) The 1–3 peaks in the HPLC profile were collected and analyzed by using MALDI-TOF mass spectrometry. The unidentified peaks are represented by *. Symbols are identical to those used in Fig. 1.

*N*-glycans of rhGAA, produced in transgenic rice cell suspension cultures containing mannosidase inhibitors, were released and analyzed using HPLC and MALDI-TOF mass spectrometry (Fig. 5). The rhGAA displayed the number 1 peak with a shoulder in the HPLC profile (the upper panel in Fig. 5A), consisting of one major Man9GlcNAc_2_ and two minor Man_7-8_GlcNAc_2_ glycans (Fig. 5B).

The rhGAA containing Man_7-9_GlcNAc_2_ glycans was used as the substrate for mannosyl-phosphorylation by rMnn14_77-935_. The reaction proceeded in the pre-determined optimal condition for a prolonged time (~24 h). The resulting HPLC profile demonstrated the generation of the number 2 and number 3 peaks (Fig. 5A), which comprise mono-mannosyl-phosphorylated (ManP-Man_7-9_GlcNAc_2_) and bis-mannosyl-phosphorylated glycans (Man_2_P_2_-Man_7-9_GlcNAc_2_), respectively. Notably, 74% of the Man_7-9_GlcNAc_2_ glycans were mannosyl-phosphorylated by the in vitro reaction using rMnn14_77-935_ and, moreover, most of the resulting mannosyl-phosphorylated glycans were bis-mannosyl-phosphorylated glycans, which can be converted to bis-phosphorylated M6P glycans with superior lysosomal targeting capability.

## Discussion

M6P glycan, a signal for lysosomal targeting, is the crucial factor determining the therapeutic efficacy of recombinant enzymes used in the treatment of lysosomal storage diseases. Glyco-engineering strategies to increase the M6P glycan contents have been designed to develop “Biobetter” therapeutic enzymes [1]. Specifically, the Callewaert group showed that glyco-engineered *Y. lipolytica* yeast produced rhGAA containing mannosyl-phosphorylated glycans, which was converted to M6P glycan using a novel uncapping enzyme [3]. Because our group also developed glyco-engineered *S. cerevisiae* expressing MPEs with strong mannosyl-phosphorylation activity [7], we attempted to produce therapeutic enzymes in these yeasts. However, the resulting secretory expression levels were disappointingly low. As an alternative method, we developed the new strategy to increase the M6P glycan content via the conjugation of glycopeptides containing M6P glycans (M6PgP) derived from glyco-engineered *S. cerevisiae* [15]. The M6PgP-conjugated rhGAA showed greatly improved lysosomal targeting and the efficient digestion of glycogen accumulated in cells from Pompe disease patients [15]. Although this strategy successfully increased the M6P glycan content, it raised quality control problems because the peptide sequences of M6PgP and their attachment sites to rhGAA cannot be controlled. Therefore, we considered in vitro mannosyl-phosphorylation using an MPE as a direct and straightforward way to increase the M6P glycan content.

 The first challenge, when attempting to establish in vitro mannosyl-phosphorylation, was to obtain a soluble recombinant MPE, as MPEs are type II membrane proteins. Because our previous study revealed strong mannosyl-phosphorylation activities of YlMpo1 and Mnn14 [7-8], they were expressed in *P. pastoris* which has the superior secretory expression capabilities. *P. pastoris* clones that secreted Mnn14 with mannosyl-phosphorylation activity were found, whereas clones that secreted YlMpo1 were not found. Because most glycosyltransferases contain the cytosolic tail, transmembrane domain, and stem region (CTS) in front of a catalytic domain, their catalytic activities are often affected by the corresponding N-terminal starting site [12]. Therefore, a series of N-terminal-deleted forms were prepared and analyzed, showing that N-terminal 76 amino acid deletion (rMnn14_77-935_) improved the activity. Here, rMnn14_77-935_ has the deletions of a cytosolic tail and a transmembrane domain (46 amino acids), as well as part of the putative stem region (30 amino acids). This suggests that the 30 amino acids in the stem region interfere with the activity of the soluble recombinant Mnn14. Further deletions beyond the 76 amino acids had a negative effect on the mannosyl-phosphorylation activity. Also, our results showed that the addition of C-terminal His-tag decreased the catalytic activity. These results suggest that rMnn14_77-935_ displayed the highest activity because it does not contain unnecessary parts.

The optimum reaction condition for the soluble rMnn14_77-935_ was established using a high-mannose type *N*-glycan (Man_8_GlcNAc_2_) as a substrate. Notably, the buffer compound has a much stronger effect on the mannosyl-phosphorylation activity than pH itself. The optimized reaction condition was applied for the mannosyl-phosphorylation of high mannose type *N*-glycans (Man_7-9_GlcNAc_2_) on rhGAA. Impressively, 74% of the glycans were bis-mannosyl-phosphorylated. This bis-form can be converted to bis-phosphorylated glycans [3, 15], which have higher affinity for MPR and better lysosomal targeting capabilities [16].

For M6P glycan synthesis, mannosyl-phosphorylation requires the following uncapping reaction to remove the outer mannose (Fig. 1B), while the mammalian GlcNAc-1-phosphotransferase reaction requires the following process to uncover the outer GlcNAc (Fig. 1A). Although the complexities of the entire process of M6P glycan synthesis are comparable, an in vitro reaction using rMnn14_47-935_ offers substantial advantages. First, the preparation of rMnn14_47-935_ is easier than that of GlcNAc-1-phosphotransferase. Because GlcNAc-1-phosphotransferase is a multisubunit enzyme with the α_2_β_2_γ_2_ arrangement, it is highly challenging to prepare a functional recombinant enzyme complex [17-18]. Secondly, MPEs found in yeasts do not appear to recognize specific proteins, whereas GlcNAc-1-phosphotransferase discriminates approximately 60 lysosomal enzymes from many non-lysosomal proteins [18]. It is essential to distinguish the degradation enzymes and confine them in the lysosome to maintain cellular integrity and homeostasis. As GlcNAc-1-phosphotransferase recognizes lysosomal enzymes in a conformation-dependent manner [18], several enzymes, including rhGAA, are poor substrates [17, 19]. In contrast, the minimal requirement for MPE substrates was reported to have at least one α(1,2)-linked mannose outside of the mannosyl-phosphorylation site [7, 20]. They appear to add mannosyl-phosphate to the mannose residues of high-mannose type *N*-glycans and *O*-mannoses on glycoproteins in order to strengthen the cell wall of the yeast [21].

In vitro mannosyl-phosphorylation using rMnn14_77-935_ is a flexible method to increase the M6P glycan contents of enzymes used as a treatment for lysosomal storage diseases. If therapeutic enzymes containing high-mannose type *N*-glycan are prepared from any production system, such as mammalian or plant cell cultures, they can serve as substrates for rMnn14_77-935_.

In summary, we generated a soluble recombinant MPE, rMnn14_77-935_, with high mannosyl-phosphorylation activity, for the first time. An in vitro reaction using rMnn14_77-935_ mannosyl-phosphorylated the glycans on rhGAA, which could then be converted to have high M6P glycans contents. These results hold promise with regard to the generation of “Biobetter” therapeutic enzymes with improved lysosomal targeting capabilities.

## Supplemental Material

Supplementary data for this paper are available on-line only at http://jmb.or.kr.
