# Supplementary material for: In Vitro N-Glycan Mannosyl-Phosphorylation of a Therapeutic Enzyme by Using Recombinant Mnn14 Produced from Pichia pastoris
Source: J Microbiol Biotechnol. 2020 Oct 28;31(1):163–70. doi: 10.4014/jmb.2010.10033 (PMC9705852; doi:10.4014/jmb.2010.10033)
Supplement: Supplementary file 1 [file jmb-31-1-163-supple.pdf]

J. Microbiol. Biotechnol.
https://doi.org/10.4014/jmb.2010.10033

Table S1. Microbial strains and plasmids used in this study

Microbial strains, plasmids	Relevant characteristics	Sources
**Strains**		
E. coli DH5α	F^- *endA1 glnV44 thi-1 recA1 relA1 gyrA96 deoR nupG purB20* φ80d*lacZ*ΔM15 Δ(*lacZYA-argF*)U169, hsdR17($r_K^- m_K^+$), λ^-	Laboratory stock
Pichia pastoris PPS9016	Host for secretory expression of recombinant Mnn14 and YlMpo1	purchased from ATUM
**Plasmids**		
pPIZαA	Vector for secretory expression of recombinant Mnn14 and YlMpo1	purchased from Thermo Fisher
YEp352-Mnn14	Template for Mnn14 gene PCR-amplication	[8]
YEp352-YlMpo1	Template for Ylmpo1 gene PCR-amplication	[7]
pPrMnn14$_{47\text{-}935}$	pPIZαA containing Mnn14$_{47\text{-}935}$	This study
pPrMnn14$_{47\text{-}935}$-H$_6$	pPIZαA containing Mnn14$_{47\text{-}935}$-H$_6$	This study
pPrMnn14$_{77\text{-}935}$-H$_6$	pPIZαA containing Mnn14$_{77\text{-}935}$-H$_6$	This study
pPrMnn14$_{107\text{-}935}$-H$_6$	pPIZαA containing Mnn14$_{107\text{-}935}$-H$_6$	This study
pPrMnn14$_{147\text{-}935}$-H$_6$	pPIZαA containing Mnn14$_{147\text{-}935}$-H$_6$	This study
pPrMnn14$_{77\text{-}935}$	pPIZαA containing Mnn14$_{77\text{-}935}$	This study
pPrMnn14$_{107\text{-}935}$	pPIZαA containing Mnn14$_{107\text{-}935}$	This study
pPrMnn14$_{47\text{-}921}$-H$_6$	pPIZαA containing Mnn14$_{47\text{-}921}$-H$_6$	This study
pPrMnn14$_{47\text{-}850}$-H$_6$	pPIZαA containing Mnn14$_{47\text{-}850}$-H$_6$	This study
pPrYlMpo1$_{36\text{-}644}$	pPIZαA containing YlMpo1$_{36\text{-}644}$	This study
pPrYlMpo1$_{36\text{-}644}$-H$_6$	pPIZαA containing YlMpo1$_{36\text{-}644}$-H$_6$	This study

Table S2. The PCR primers used for vector construction

Primers	Sequences[a]
M14_47F	CCGGCCGTCTCGGATCGGTACCGACGGTAACTGGAAGTCATTC
M14_935R	GAGATGAGTTTTTGTTCTAGACTAATATTTTTGGTCTGAACCAA
M14_935HR	GATGAGTTTTTGTTCTAGACTA*ATGATGATGATGATGATG*ATATTTTTGGTCTGAAC
M14_77F	CCGGCCGTCTCGGATCGGTACCTCAAAAGACAACGTAGCC
M14_77-935HR	GAGATGAGTTTTTGTTCTAGACTA*ATGATGATGATGATGATG*ATA
M14_107F	CCGGCCGTCTCGGATCGGTACCCTCACTGTGAAAATGGGTCC
M14_147F	CCGGCCGTCTCGGATCGGTACCGATTCCAATGAATACGCATTTTC
M14_921HR	GAGATGAGTTTTTGTTCTAGACTA*ATGATGATGATGATGATG*ATTATCACCAAAGAGA
M14_850HR	GAGATGAGTTTTTGTTCTAGACTA*ATGATGATGATGATGATG*TTCGTATATATATATG
YM_36F	CCGGCCGTCTCGGATCGGTACCAAGCTGCCCGACGGAGTCAAG
YM_644R	GATGAGTTTTTGTTCTAGACTACTCAAACTCCTCGCGAATC
YM_644HR	GATGAGTTTTTGTTCTAGACTA*ATGATGATGATGATGATG*CTCAAACTCCTCGCG

[a] Sequences for EZ-Fusion and Electra cloning are underlined while those for His-tag addition are represented by italic characters.

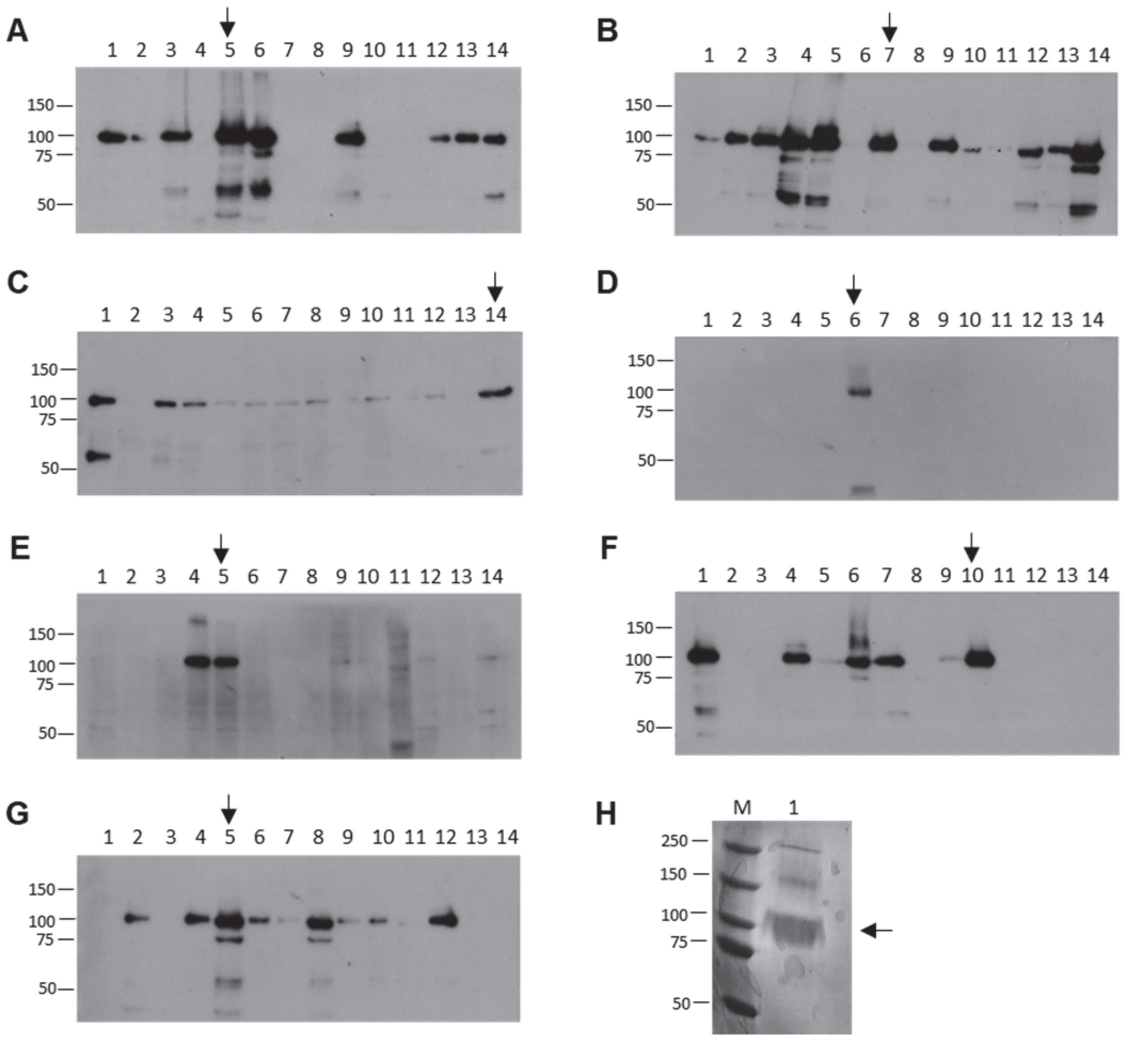

Fig. S1. Detection of recombinant Mnn14 proteins. (A-G) To select the best clones, the amounts of Mnn14 proteins in the culture supernatants were analyzed by Western blots using an anti-Mnn14 antibody; rMnn14$_{47\text{-}935}$ (A), rMnn14$_{47\text{-}935}$-H$_6$ (B), rMnn14$_{77\text{-}935}$-H$_6$ (C), rMnn14$_{107\text{-}935}$-H$_6$ (D), rMnn14$_{147\text{-}935}$-H$_6$ (E), rMnn14$_{77\text{-}935}$ (F), and rMnn14$_{107\text{-}935}$ (G). The arrows indicate the selected clones. (H) SDS-PAGE analysis of the purified rMnn14$_{77\text{-}935}$. M, marker; lane 1, purified rMnn14$_{77\text{-}935}$.
